# Effectiveness of eHealth Self-management Interventions in Patients With Heart Failure: Systematic Review and Meta-analysis

**DOI:** 10.2196/38697

**Published:** 2022-09-26

**Authors:** Siru Liu, Jili Li, Ding-yuan Wan, Runyi Li, Zhan Qu, Yundi Hu, Jialin Liu

**Affiliations:** 1 Department of Biomedical Informatics Vanderbilt University Medical Center Nashville, TN United States; 2 West China School of Medicine Sichuan University Chengdu China; 3 College of Computer Science Sichuan University Chengdu China; 4 School of Data Science Fudan University Shanghai China; 5 Department of Medical Informatics West China Hospital Sichuan University Chengdu China

**Keywords:** heart failure, eHealth, self-management, systematic review, cardiology, cardiovascular, morbidity

## Abstract

**Background:**

Heart failure (HF) is a common clinical syndrome associated with substantial morbidity, a heavy economic burden, and high risk of readmission. eHealth self-management interventions may be an effective way to improve HF clinical outcomes.

**Objective:**

The aim of this study was to systematically review the evidence for the effectiveness of eHealth self-management in patients with HF.

**Methods:**

This study included only randomized controlled trials (RCTs) that compared the effects of eHealth interventions with usual care in adult patients with HF using searches of the EMBASE, PubMed, CENTRAL (Cochrane Central Register of Controlled Trials), and CINAHL databases from January 1, 2011, to July 12, 2022. The Cochrane Risk of Bias tool (RoB 2) was used to assess the risk of bias for each study. The Grading of Recommendations, Assessment, Development, and Evaluation (GRADE) criteria were used to rate the certainty of the evidence for each outcome of interest. Meta-analyses were performed using Review Manager (RevMan v.5.4) and R (v.4.1.0 x64) software.

**Results:**

In total, 24 RCTs with 9634 participants met the inclusion criteria. Compared with the usual-care group, eHealth self-management interventions could significantly reduce all-cause mortality (odds ratio [OR] 0.83, 95% CI 0.71-0.98, *P*=.03; GRADE: low quality) and cardiovascular mortality (OR 0.74, 95% CI 0.59-0.92, *P*=.008; GRADE: moderate quality), as well as all-cause readmissions (OR 0.82, 95% CI 0.73-0.93, *P*=.002; GRADE: low quality) and HF-related readmissions (OR 0.77, 95% CI 0.66-0.90, *P*<.001; GRADE: moderate quality). The meta-analyses also showed that eHealth interventions could increase patients’ knowledge of HF and improve their quality of life, but there were no statistically significant effects. However, eHealth interventions could significantly increase medication adherence (OR 1.82, 95% CI 1.42-2.34, *P*<.001; GRADE: low quality) and improve self-care behaviors (standardized mean difference –1.34, 95% CI –2.46 to –0.22, *P*=.02; GRADE: very low quality). A subgroup analysis of primary outcomes regarding the enrolled population setting found that eHealth interventions were more effective in patients with HF after discharge compared with those in the ambulatory clinic setting.

**Conclusions:**

eHealth self-management interventions could benefit the health of patients with HF in various ways. However, the clinical effects of eHealth interventions in patients with HF are affected by multiple aspects, and more high-quality studies are needed to demonstrate effectiveness.

## Introduction

Heart failure (HF), a major global public health concern, is a common clinical syndrome caused by cardiac structural or functional impairment [1]. The global prevalence of HF was estimated at 64.34 million cases, and the global economic burden of HF was roughly calculated at US $346.17 billion. Global expenditure related to HF is expected to increase to approximately US $400 billion by 2030 [2]. Since HF cannot be completely cured, it has a major impact on quality of life and requires long-term management [3-5]. The management of patients with HF is a complex issue. Even when the patient is clinically stable, the quality of life is reduced due to dyspnea, depression, fatigue, and cognitive impairment [6]. The symptom burden of patients with HF prevents them from maintaining adequate social life and roles, participating in social activities, and maintaining relationships [7].

Self-management is a dynamic, iterative process in which patients need to employ multidimensional strategies to meet their self-needs for coping with chronic illness in their daily lives [8,9]. Self-management is an effective way to improve the outcome of chronic disease and an important part of the treatment of patients with chronic diseases [10]. Successful self-management requires the active participation of individuals, families, and health care providers [11]. Self-management has been demonstrated to improve clinical outcomes and health-related quality of life, while reducing health care utilization and costs [12-14].

eHealth, as broadly defined, refers to a variety of information and communication technologies used to deliver health care services [15,16]. The use of eHealth interventions to deliver health care services can reduce or eliminate some of the barriers to face-to-face treatment, improve access to treatment, reduce waiting times, and be more cost-effective than face-to-face interventions [17-19]. The World Health Organization (WHO) states that to improve health and reduce health inequities, a rigorous evaluation of eHealth is necessary to generate evidence and facilitate the appropriate integration and use of technology [20]. In addition, the WHO and the International Telecommunication Union launched the National eHealth Strategy Toolkit, which is a comprehensive and practical guide to help all governments, along with their ministries, departments, and agencies, to adapt to suit their own circumstances, goals, and vision in achieving eHealth [21].

There is a large body of research demonstrating the effectiveness of eHealth self-management interventions for chronic diseases [22,23]. eHealth self-management supports patients’ empowerment to better manage HF and improve quality of life [24]. Furthermore, eHealth applications and systems could help to reduce the rehospitalization rate of patients with HF and lower the cost of treatment [25].

However, previous research has not specifically focused on the impact of eHealth in self-management. Although eHealth self-management interventions have been used in patients with HF, the results of some systematic reviews have been inconsistent [4,26]. Furthermore, there is a lack of understanding of the effectiveness and influencing factors of eHealth self-management approaches.

To fill these gaps, the aim of this study was to perform a systematic review and meta-analysis of the effectiveness of eHealth self-management interventions in patients with HF. This review will provide a reference for the clinical application and better understanding of the possible benefits of eHealth self-management interventions for patients with HF. These findings will further identify gaps and inform the development of future eHealth interventions.

## Methods

### Search Strategy

This systematic review and meta-analysis was performed following the PRISMA (Preferred Reporting Items for Systematic Reviews and Meta-Analyses) guidelines [27]. We searched the PubMed, EMBASE, CENTRAL (Cochrane Central Register of Controlled Trials), and CINAHL databases from January 1, 2011, to July 12, 2022, to identify randomized controlled trials (RCTs) that provide eHealth self-management interventions for HF patients. We used combined search terms such as (heart failure OR cardiac failure OR heart decompensation) AND (self-management OR self care OR self-administration OR self-medication) AND (Telemedicine OR mobile health OR eHealth OR m-health) AND (randomized controlled trial OR randomization OR randomly). Screening titles/abstracts and full-text evaluation of the articles were managed in a database created with Rayyan software [28]. The full protocol of this systematic review and meta-analysis has been registered in PROSPERO (CRD42021246973).

### Selection Criteria

Inclusion and exclusion criteria strictly followed the Participants-Intervention-Comparison-Outcome (PICO) framework [29]: (1) participants were defined as adults (aged≥18 years) who had been diagnosed with HF (studies in children or adolescents were excluded); (2) interventions consisted of self-management tools including at least one eHealth component (eg, mobile apps), with traditional interventions that did not use any technical support excluded, such as face-to-face meetings; (3) the comparison was HF patients versus usual care; and the (4) outcomes included patient and process outcomes. Patient outcomes are measures directly related to the disease and were considered as the primary outcomes in our study, including all-cause readmission rate, all-cause mortality, HF readmission, and cardiovascular mortality. Process outcomes are measures related to patients’ behavior, which were considered as the secondary outcomes in our study, including quality of life, self-care behaviors, HF knowledge, and medication adherence. In addition, only RCTs or cluster RCTs reporting one or more selected outcomes were included. Reviews, editorials, protocols, and non-English papers were excluded.

### Data Extraction

The following characteristics were extracted from studies that met the inclusion criteria: name of the first author, publication year, country, type of eHealth technologies, target of eHealth intervention, duration of intervention, patient demographics, number of participants, recruitment setting, outcomes, descriptions of the control, and the intervention. All outcome data used for the meta-analysis were extracted by the same two independent reviewers (J Li and DW). The synthesis of the results and data charting were performed independently by two researchers (SL and J Li). Disagreements were discussed and consulted with a third reviewer (J Liu) to reach consensus.

### Risk of Bias Assessment

We used the Cochrane Risk of Bias tool (RoB 2) to assess the risk of bias for each study [30]. This tool assesses 5 domains to address different types of bias: randomization process, deviations from the intended interventions, missing outcome data, measurement of the outcome, and selection of the reported result. Each component includes a low risk of bias, some concerns, or high risk of bias. Risk of bias assessment was performed independently by two reviewers with consultation of the third reviewer when necessary.

### Certainty of Evidence

The Grading of Recommendations, Assessment, Development, and Evaluation (GRADE) approach was used to rate the certainty of evidence for each outcome of interest [31]. This approach rates the risk of bias, inconsistency, indirectness, imprecision, and other considerations (eg, publication bias) as “high,” “moderate,” “low,” or “very low.” The GRADE assessment was completed with the GRADEpro Guideline Development Tool.

### Statistical Analyses

We performed meta-analyses using Review Manager [32] (RevMan v.5.4; Cochrane Training) and R (v.4.1.0 x64). The Mantel-Haenszel odds ratio (OR) was used as the effect size for dichotomous outcomes and the standardized mean difference (SMD) was used to calculate the intervention effect for continuous outcomes. Heterogeneity was then analyzed using the Cochran *Q* test and the *I^2^* statistic. For the *Q* test, *P*<.10 was considered to indicate statistically significant heterogeneity [33]. According to Cochrane Handbook guidelines [34], the interpretation of the *I^2^* statistic was as follows: 0% to 40% may represent not important heterogeneity, 30% to 60% may represent moderate heterogeneity, 50% to 90% may represent substantial heterogeneity, and 75% to 100% may represent considerable heterogeneity. The fixed-effects model was used for outcomes with low heterogeneity and the random-effects model was used when the heterogeneity was significantly high. To measure the publication bias, we constructed funnel plots and performed the Egger test for all primary outcomes to determine the significance of potential asymmetry [35]. The threshold of a significant *P* value was .05. A sensitivity analysis was performed to check the robustness of the pooled results using the leave-one-out approach [36]. 

## Results

### Search Outcomes

**Figure 1:**
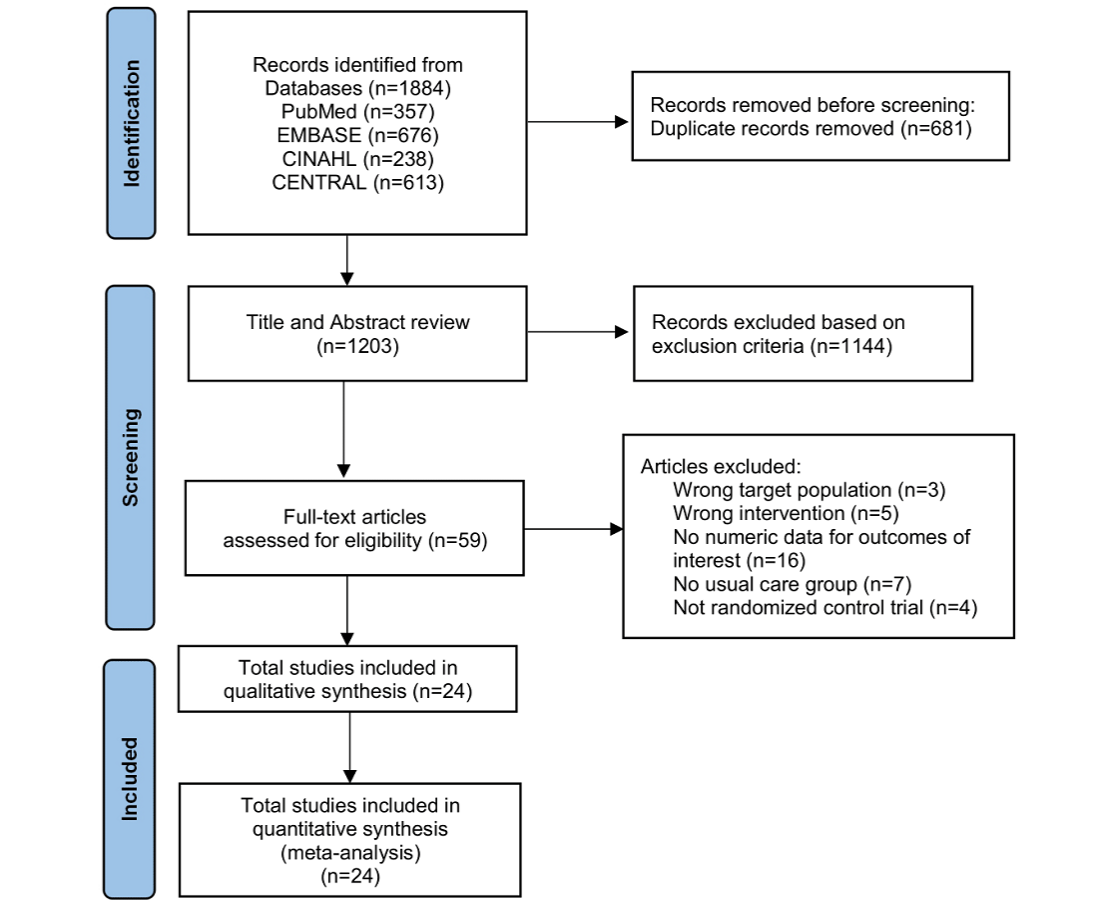
PRISMA (Preferred Reporting Items for Systematic Reviews and Meta-Analyses) flow diagram for selection and inclusion of the studies via databases. Latest search date: July 12, 2022.

A total of 1884 articles were retrieved in the literature search. After eliminating duplicates, 1203 titles and abstracts were screened in relation to the inclusion/exclusion criteria. Of these, 1144 articles were excluded and a total of 59 articles were subject to full-text review. Finally, 24 articles were included in the systematic review and meta-analysis (Figure 1).

### Risk of Bias and Quality Assessment

**Figure 2:**
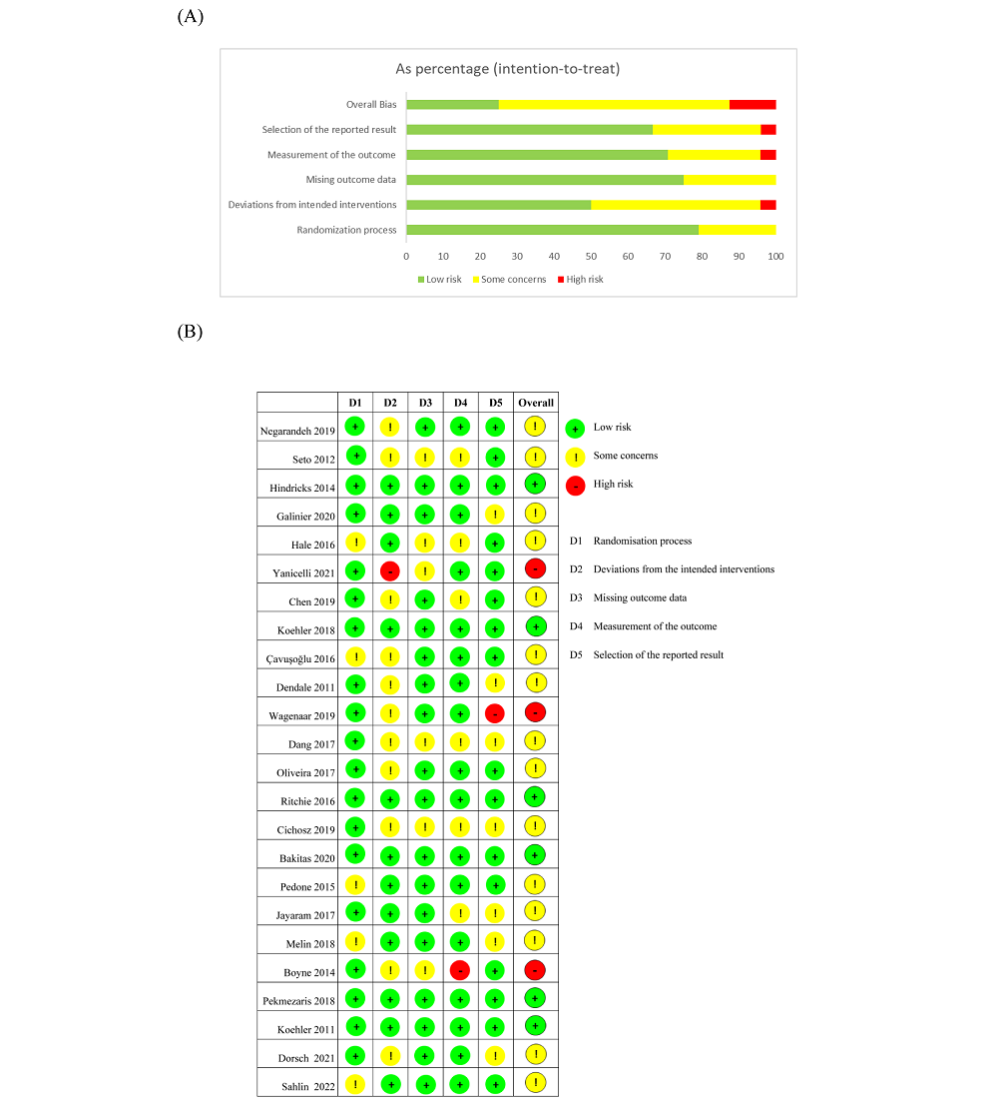
Quality assessment. (A) Each risk of bias domain presented as a percentage across all included studies. (B) Each risk of bias domain for each included study.

The results of risk of bias assessment are summarized in Figure 2. The majority of studies showed a low risk of bias for the randomization process, and only 5 studies were judged to have some concerns due to lack of detailed information about randomization. The studies could not have been blinded to participants considering the nature of the intervention and 11 studies were judged to have some concerns regarding deviations from the intended interventions. One study was determined as “high risk” for the category of measurement of the outcome because all questionnaire scores were patient-reported, which may have possibly affected the outcome [37]. In addition, one study did not report all of the outcomes according to the trial registry record, resulting in a judgment of “high risk” regarding selection of the reported result [38]. For studies included in the meta-analysis, the overall bias was low in 6 studies (25%), with some concerns in 15 studies (63%) and high bias in 3 studies (13%).

### Study Characteristics

A total of 9634 participants with HF were included in the 26 (22+2×2) RCTs, with 4820 patients allocated to the eHealth group and 4814 to the usual-care group. Since two studies performed a three-group parallel-randomized trial and reported their results separately [38,39], we divided each of the three-group trials into two RCTs. These studies were performed in 20 different countries, with the largest number of studies performed in the United States (7 studies). Most (15 studies) were multicenter studies, while the other 11 RCTs recruited patients from a single center. The proportion of male HF patients ranged from 46.8% to 83% in the eHealth groups and from 30.2% to 93% in the usual-care groups. The New York Heart Association classification, which classifies patients into one of four categories based on their limitations during physical activity and has been used clinically to determine trial eligibility, was reported in 24 studies. The basic characteristics of the included studies are presented in Table 1, whereas the characteristics of participants and details of interventions are summarized in Multimedia Appendix 1.

**Table 1:** Basic characteristics of studies included in the meta-analysis.

Reference (year)	Country	Type of eHealth technologies	Target of eHealth intervention	Duration of intervention	Recruitment	Setting
Negarandeh et al [40] (2019)	Iran	Telephone	Education	2 months	Single center	After discharge
Seto et al [41] (2012)	Canada	Designed telemedical system	Monitoring	6 months	Single center	Ambulatory clinic
Hindricks et al [42] (2014)	Australia, Europe^a^, and Israel	Designed telemedical system	Monitoring	12 months	Multicenter (36 sites)	No information
Galinier et al [43] (2020)	France	Telephone+designed telemedical system	Monitoring+education	18 months	Multicenter (38 sites)	After discharge (26.4%), hospitalized (73.6%)
Hale et al [44] (2016)	United States	Designed telemedical system	Reminders	3 months	Multicenter (2 sites)	No information
Yanicelli et al [45] (2021)	Argentina	Mobile or tablet app	Monitoring+education	3 months	Single center	Ambulatory clinic
Chen et al [39] (2019)^b^	China	Mobile text message	Education+reminders	6 months	Single center	After discharge
Chen et al [39] (2019)^b^	China	Telephone	Education+reminders	6 months	Single center	After discharge
Koehler et al [46] (2018)	Germany	Designed telemedical system	Monitoring+education	365-393 days	Multicenter (200 sites)	After discharge
Çavuşoğlu et al [47] (2016)	Turkey	Telephone	Education	6 months	Multicenter (10 sites)	After discharge
Dendale et al [48] (2011)	Belgium	Designed telemedical system	Monitoring	6 months	Multicenter (7 sites)	After discharge
Wagenaar et al [38] (2019)^b^	Netherlands	Internet website	Education	12 months	Multicenter (9 sites)	Ambulatory clinic
Wagenaar et al [38] (2019)^b^	Netherlands	Designed telemedical system	Monitoring	12 months	Multicenter (9 sites)	Ambulatory clinic
Dang et al [49] (2017)	United States	Designed telemedical system	Monitoring	3 months	Single center	Ambulatory clinic
Oliveira et al [50] (2017)	Brazil	Telephone	Education	4 months	Single center	Ambulatory clinic
Ritchie et al [51] (2016)	United States	Designed telemedical system	Mixed interventions	1 months	Single center	After discharge
Cichosz et al [52] (2019)	Denmark	Designed telemedical system	Monitoring	12 months	Multicenter (3 sites)	Ambulatory clinic
Bakitas et al [53] (2020)	United States	Telephone	Mixed interventions	4 months	Multicenter (2 sites)	Ambulatory clinic, hospitalized
Pedone et al [54] (2015)	Italy	Telephone+designed telemedical system	Monitoring	6 months	Single center	Ambulatory clinic after discharge
Jayaram et al [55] (2017)	United States	Designed telemedical system	Monitoring	6 months	Multicenter (33 sites)	After discharge
Melin et al [56] (2018)	Sweden	Mobile or tablet app	Monitoring+education	6 months	Multicenter (3 sites)	After discharge, hospitalized
Boyne et al [37] (2014)	Netherlands	Designed telemedical system	Monitoring+education	12 months	Multicenter (3 sites)	Ambulatory clinic
Pekmezaris et al [57] (2018)	United States	Designed telemedical system	Monitoring	3 months	Single center	After discharge
Koehler et al [58] (2011)	Germany	Designed telemedical system	Monitoring	Median 26 months (range 12-28 months)	Multicenter (165 sites)	Ambulatory clinic
Dorsch et al [59] (2021)	United States	Mobile or tablet app	Mixed interventions	3 months	Single center	After discharge, hospitalized
Sahlin et al [60] (2022)	Sweden	Designed telemedical system	Monitoring+education	8 months	Multicenter (7 sites)	Ambulatory clinic

^a^Europe includes only Austria, Czech Republic, Denmark, Germany, and Latvia.

^b^Two types of eHealth interventions employed in a three-group parallel randomized controlled trial design.

### Intervention

These studies used various types of eHealth technologies for the intervention (Table 1). Of these, 14 studies used a designed telemedical system; 5 studies used the telephone; 3 studies used a mobile phone or tablet app; 2 studies used an internet website and text messages, respectively; and 2 studies used a combination of telephone with a designed telemedical system intervention. The duration of the intervention ranged from 1 month to 28 months (17 studies had interventions lasting≥6 months). The target of the intervention was classified into three types: education, monitoring, and reminders. The mixed targets were defined as the presence of the three types in one study. Educational interventions were defined as interventions that aimed at improving the HF knowledge available through educational programs and instructions. Reminder interventions were defined as interventions that prompted patients to do something (eg, take medication on time) and could be delivered through a designed telemedical system, mobile text messages, or telephone calls. Monitoring interventions were defined as interventions that transmitted vital signs or symptoms (eg, weight, blood pressure, or heart rate) to an external telemedicine center, which may lead to lasting improvements in behaviors. The eHealth intervention varied regarding intensity and the extent of the human component, although most trials provided the eHealth intervention every day (Multimedia Appendix 1).

### Primary Outcomes

#### Effects of eHealth Interventions on All-Cause Mortality

**Figure 3:**
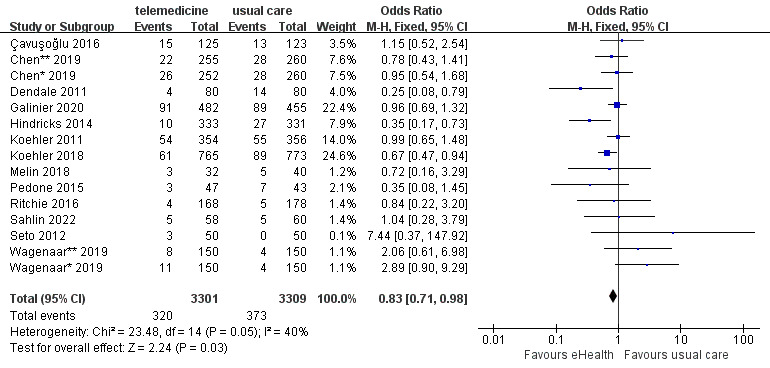
Forest plot of the effects of eHealth interventions on all-cause mortality.

A total of 15 studies reported all-cause mortality data. The 15 studies included 6610 participants and showed moderate heterogeneity (*P*=.05; *I^2^*=40%). The analysis showed that all-cause mortality was significantly lower in the eHealth intervention group than in the control group (OR 0.83, 95% CI 0.71-0.98, *P*=.03; GRADE: low quality) (Figure 3).

#### Effects of eHealth Interventions on Cardiovascular Mortality

**Figure 4:**
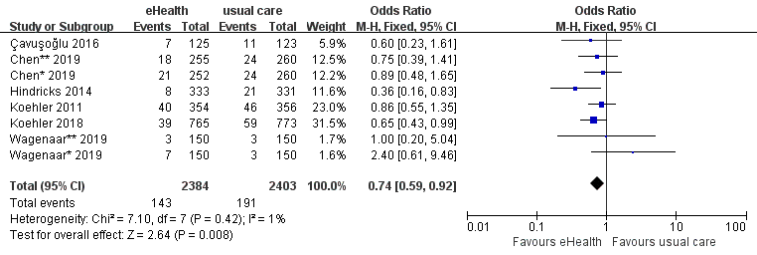
Forest plot of the effects of eHealth interventions on cardiovascular mortality.

A total of 8 studies reported cardiovascular mortality data. The 8 studies included 4787 participants and had low heterogeneity (*P*=.42; *I^2^*=1%). The analysis showed that cardiovascular mortality was significantly lower in the eHealth intervention group than in the control group (OR 0.74, 95% CI 0.59-0.92, *P*=.008; GRADE: moderate quality) (Figure 4).

#### Effects of eHealth Interventions on All-Cause Readmission

**Figure 5:**
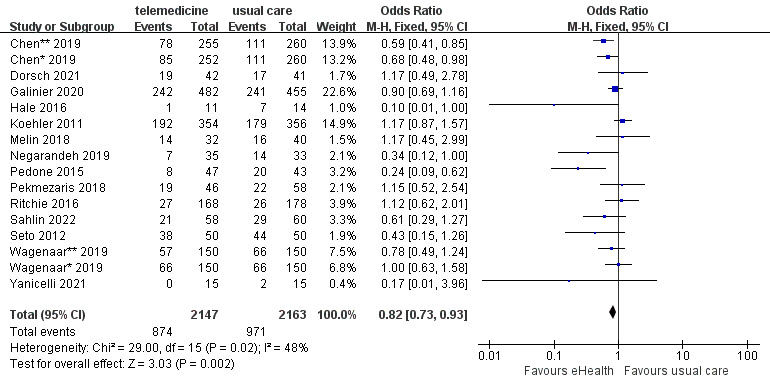
Forest plot of the effects of eHealth interventions on all-cause readmission.

A total of 16 studies reported all-cause readmission data. The 16 studies included 4310 participants and had moderate heterogeneity (*P*=.02; *I^2^*=48%). The analysis showed that all-cause readmission was significantly lower in the eHealth intervention group than in the control group (OR 0.82, 95% CI 0.73-0.93, *P*=.002; GRADE: low quality) (Figure 5).

#### Effects of eHealth Interventions on HF-Related Readmission

**Figure 6:**
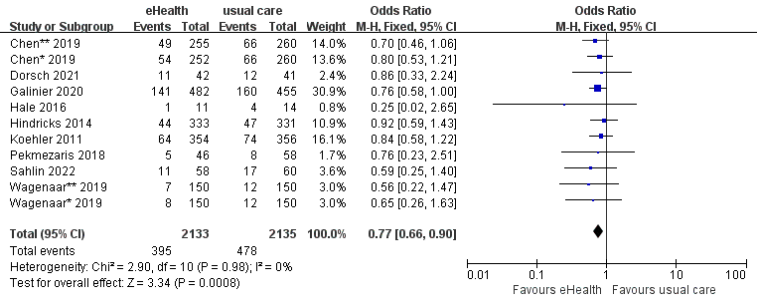
Forest plot of the effects of eHealth interventions on heart failure–related readmission.

A total of 11 studies reported HF-related readmission data. The 11 studies included 4268 participants and had no heterogeneity (*P*=.98; *I^2^*=0%). The analysis showed that HF-related readmission was significantly lower in the eHealth intervention group than in the control group (OR 0.77, 95% CI 0.66-0.90, *P*<.001; GRADE: moderate quality) (Figure 6).

### Secondary Outcomes

#### Effects of eHealth Interventions on HF Knowledge

**Figure 7:**
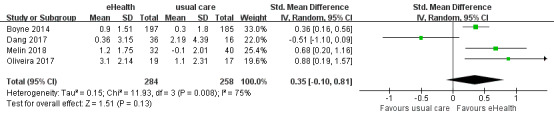
Forest plot of the effects of eHealth interventions on heart failure knowledge.

A total of 4 studies reported HF knowledge data, 3 of which used the 15-item Dutch HF Knowledge Scale (DHFKS) [61]. One study used a modified questionnaire containing 14 questions, which has proven to be an adequate tool to evaluate the knowledge of Brazilian HF patients [62]. Higher scores across both scales indicate that the patients have more knowledge of HF. The analysis demonstrated an improvement in HF knowledge among patients in the eHealth intervention group compared with that in the control group. However, there was no significant difference between the eHealth and usual-care groups (SMD 0.35, 95% CI –0.10 to 0.81, *P*=.13; GRADE: very low quality), with significantly high heterogeneity between studies (*P*=.008; *I^2^*=75%) (Figure 7).

#### Effects of eHealth Interventions on Quality of Life

**Figure 8:**
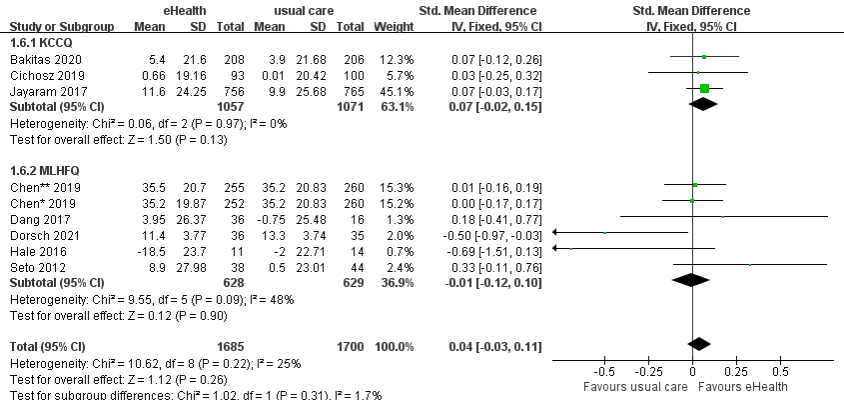
Forest plot of the effects of eHealth interventions on quality of life. KCCQ: Kansas City Cardiomyopathy Questionnaire; MLHFQ: Minnesota Living with Heart Failure Questionnaire.

A total of 9 studies reported general quality of life data. Three of these studies used the HF-specific 23-item Kansas City Cardiomyopathy Questionnaire (KCCQ), in which higher scores indicate better quality of life [63]. Five of these studies used the Minnesota Living with Heart Failure Questionnaire (MLHFQ), which consists of 21 questions using a 6-point Likert scale [64]. Since lower scores of the MLHFQ indicate a higher quality of life, we calculated the change using the baseline score minus the final score. The analysis demonstrated an improvement in quality of life among patients in the eHealth intervention group compared with that of the control group. However, there was no significant difference between the eHealth and usual-care groups (SMD 0.04, 95% CI –0.03 to 0.11, *P*=.26; GRADE: moderate quality), with low heterogeneity among the 9 studies (*P*=.22; *I^2^*=25%) (Figure 8).

#### Effects of eHealth Interventions on Medication Adherence

**Figure 9:**
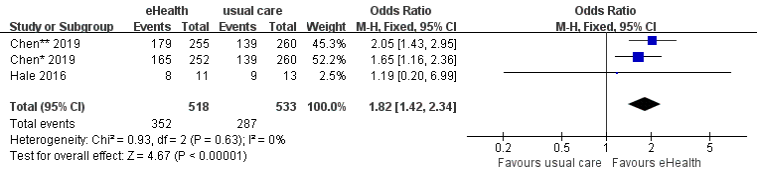
Forest plot of the effects of eHealth interventions on medication adherence.

A total of 3 studies reported medication adherence data. The analysis demonstrated a significant improvement in medication adherence among patients in the eHealth intervention group compared with the control group (OR 1.82, 95% CI 1.42-2.34, *P*<.001; GRADE: low quality), with no heterogeneity among 3 studies (*P*=.63; *I^2^*=0%) (Figure 9).

#### Effects of eHealth Interventions on Self-care Behaviors

**Figure 10:**
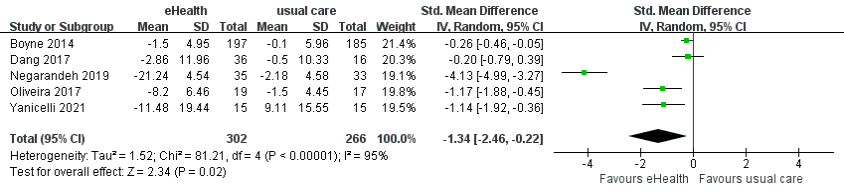
Forest plot of the effects of eHealth interventions on self-care behaviors.

A total of 3 studies reported self-care behavior data. Four of these studies used the European Heart Failure Self-Care Behavior Scale (EHFSC) [65]. Two of these studies used the EHFSC revised to a 9-item scale (EHFScB-9) [66], and 2 used the self-care of heart failure index (SCHFI), which comprises three subscales [67]. Three of the 8 studies had imperfect data and therefore were not included in this meta-analysis. The analysis demonstrated a significant improvement in self-care behaviors among patients in the eHealth intervention group compared with the control group (SMD –1.34, 95% CI –2.46 to –0.22, *P*=.02; GRADE: very low quality), with significantly high heterogeneity among 5 studies (*P*<.001; *I^2^*=95%) (Figure 10).

### Subgroup Analyses

Since the inclusion criteria of the trials were different, which contributed to the heterogeneity of studies, subgroup analyses of primary outcomes regarding the enrolled population setting (after discharge or ambulatory clinic) were performed and the results are summarized in Multimedia Appendix 2. Compared with patients in an ambulatory clinic setting, the eHealth intervention showed a larger effect in patients with HF after discharge on the reduction of all-cause mortality (OR 0.73, 95% CI 0.58-0.93, *P*=.01), cardiovascular mortality (OR 0.71, 95% CI 0.53-0.95, *P*=.02), all-cause readmission (OR 0.70, 95% CI 0.56-0.87, *P*=.001), and HF-related readmission (OR 0.75, 95% CI 0.56-1.00, *P*=.05) than the usual-care group. The results showed that the effects of an eHealth intervention may vary in certain population settings.

### Sensitivity Analysis and Publication Bias

Sensitivity analysis was performed for the primary outcomes of meta-analyses, including all-cause mortality, cardiovascular mortality, all-cause readmission rate, and HF-related hospitalizations, using the leave-one-out approach. The direction and magnitude of the combined estimates did not vary markedly with the exclusion of individual studies, indicating the reliability of the findings (Multimedia Appendix 3). The results showed no publication bias in the primary outcomes. No publication bias was detected for any of the primary outcomes. The funnel plots and linear regression test results of funnel plot asymmetry are shown in Multimedia Appendix 4.

## Discussion

### General Findings

There is a reasonable amount of original research exploring the effects of eHealth interventions in HF. However, among the 1884 articles we identified, only 24 met the inclusion criteria for this systematic review. The pooled results suggest that eHealth self-management interventions can improve primary and secondary outcomes in patients with HF.

### Implementation Challenges

#### Overview

Although eHealth self-management interventions have the potential to improve chronic disease management, successful implementation in routine clinical practice is rare [68,69]. The main challenges in implementing and utilizing eHealth include user acceptance, standards and interoperability, regulations, ensuring cost-effectiveness and sustainability, and the organization and implementation environment, as discussed in turn below.

#### eHealth User Acceptance

End-user acceptance of eHealth products is the key to a successful eHealth program, which is influenced by many factors, although eHealth usability and user training seem to be more important. eHealth products need to improve the understanding of the design and development process from the user’s perspective, and focus on developing detailed representations of the user’s needs and wants [70-72]. Furthermore, the process of eHealth interventions is complex, which requires active participation, cooperation, and familiarity with the associated processes by all involved [11]. Through eHealth self-management, health care providers can connect more closely with patients, identify problems earlier, provide guidance, and improve patient compliance.

#### Standards and Interoperability

A successful eHealth self-management implementation requires unified integration with electronic health record (EHR) and clinical workflows to enable secure and fast access to patient data and information at various locations [73]. This requires interoperability within EHRs, as well as interoperability between EHR and eHealth self-management systems. Ultimately, intelligent eHealth self-management could reduce phone calls and paperwork by automating data exchange. However, eHealth self-management and EHR systems often lack interoperability and standards for clinical data exchange [74,75].

#### eHealth Regulations

There is also a lack of eHealth regulations, which is a major barrier to the development of eHealth [76]. The most common regulations used in eHealth implementation are national data and privacy protection laws and regulations, as well as national EHR and health financing legislation [77]. Health financing legislation is critical to ensure the continued sustainability and support of eHealth. National health service laws also provide guidelines for the use and implementation of eHealth [78].

#### Cost-Effectiveness and Sustainability

The cost-effectiveness and sustainability of eHealth are key factors that directly affect the successful implementation of eHealth in practice [70]. Many of the costs relate to the development of the eHealth product, deployment, training, product iterations, services, or ongoing maintenance costs of the technology. eHealth self-management programs should ensure cost-effectiveness and sustainability of the program.

#### Organization and Implementation Environment

The hospital’s management capacity, human resources, and implementation environment are key obstacles to the successful implementation of eHealth [79]. In addition, eHealth technologies also need to fit the organization. The information literacy of patients and easy access to high-speed internet are conducive to the implementation of eHealth [80,81]. eHealth applications should be implemented on top of existing organization-centric and process-controlled systems [82]. This will benefit the efficiency and effectiveness of eHealth solutions and health care services, as well as support the development process and change in management.

### eHealth Self-management in HF

eHealth self-management is a rapidly growing area of HF management, and considerable research is still needed to promote the widespread use of eHealth self-management and enhance its clinical outcomes. Future research should investigate how to expand the content of eHealth self-management tools while adapting to different HF patients. There is a need for a cost-benefit analysis of eHealth self-management, and how to most effectively integrate eHealth self-management into workflows and deploy it in different settings. Further work is needed to understand how eHealth self-management can help with the transformation of care delivery models and how to combine eHealth self-management with a workflow-oriented quality improvement program.

### Limitations

Although this systematic review and associated meta-analyses showed that eHealth self-management interventions benefit patients with HF, this study has several limitations. First, the included studies varied in participant demographics, types of HF, sample size, assessment tools, period of interventions, and type of eHealth interventions, which can lead to heterogeneity and bias. Furthermore, reliance on self-reports and the use of questionnaires to score outcomes may lead to bias in self-reported data. Therefore, the results of this systematic review should be interpreted with caution. Second, participants were recruited at a single site or concentrated in a single population in some cases; thus, the resulting sample was not representative of the entire HF patient population, which would limit the generalizability of the findings. Finally, we only searched for literature in English, excluding other languages. This may limit the retrieval of non-English but related papers.

### Conclusion

In this systematic review, we performed a literature search and provided a comprehensive overview of eHealth self-management in patients with HF. We observed that eHealth self-management can support the health of HF patients in many ways. However, the effectiveness of eHealth in the self-management of patients with HF is affected by multiple aspects and more clinical studies are needed to prove its effectiveness.

## Appendix

Multimedia Appendix 1 Basic characteristics of the studies.

Multimedia Appendix 2 Subgroup analyses for primary outcomes.

Multimedia Appendix 3 Sensitivity analysis of the influence of each study on the pooled estimates.

Multimedia Appendix 4 Funnel plots of all-cause mortality.

## Abbreviations

CENTRAL: Cochrane Central Register of Controlled Trials
DHFKS: Dutch Heart Failure Knowledge Scale
EHFSC: European Heart Failure Self-Care Behavior Scale
EHFScB-9: 9-item European Heart Failure Self-Care Behavior Scale
EHR: electronic health record
GRADE: Grading of Recommendations, Assessment, Development and Evaluation
HF: heart failure
KCCQ: Kansas City Cardiomyopathy Questionnaire
MLHFQ: Minnesota Living with Heart Failure Questionnaire
OR: odds ratio
PICO: Participants-Intervention-Comparison-Outcome
PRISMA: Preferred Reporting Items for Systematic Reviews and Meta-Analyses
RCT: randomized controlled trial
RoB 2: Cochrane Risk of Bias tool
SCHFI: self-care of heart failure index
SMD: standardized mean difference
WHO: World Health Organization
