# Supplementary material for: Effectiveness of eHealth Self-management Interventions in Patients With Heart Failure: Systematic Review and Meta-analysis
Source: J Med Internet Res. 2022 Sep 26;24(9):e38697. doi: 10.2196/38697 (PMC9555330; doi:10.2196/38697)

**Multimedia Appendix 2**

Forest plots of the subgroup analyses of the setting of patients with HF (after discharge or ambulatory clinic)

Figure S1. Forest plot of the subgroup analysis of the effects of eHealth intervention on all-cause mortality.


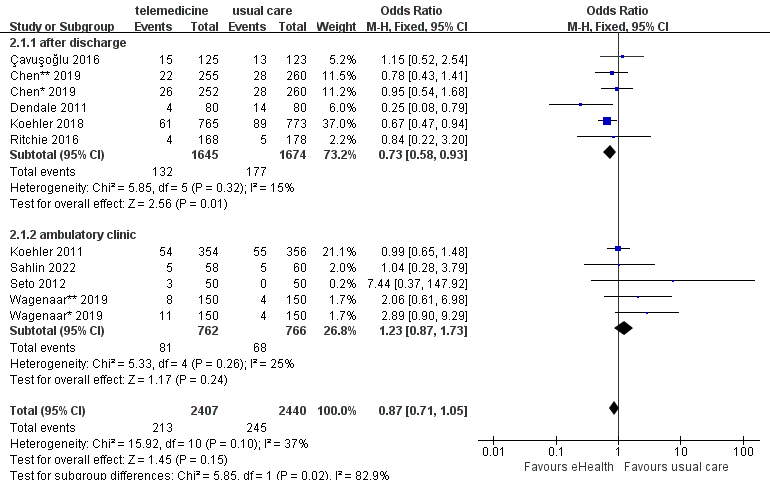


Figure S2. Forest plot of the subgroup analysis of the effects of eHealth intervention on cardiovascular mortality.


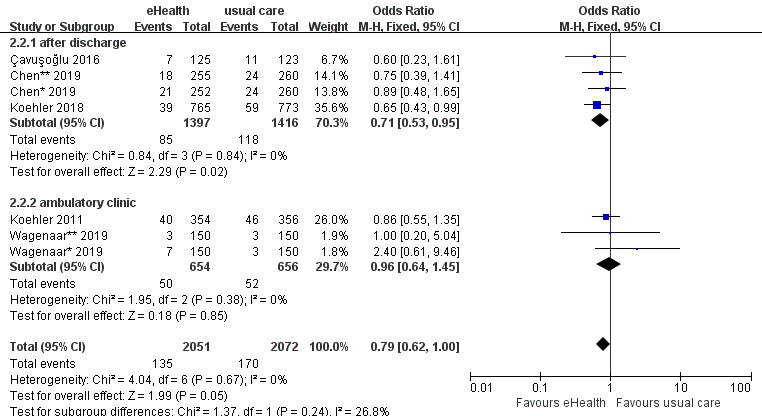


Figure S3. Forest plot of the subgroup analysis of the effects of eHealth intervention on all-cause readmission.


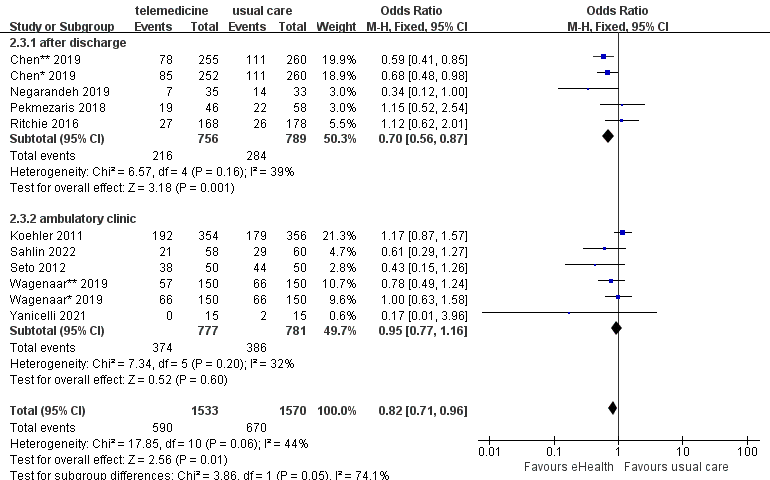


Figure S4. Forest plot of the subgroup analysis of the effects of eHealth intervention on HF-related readmission.


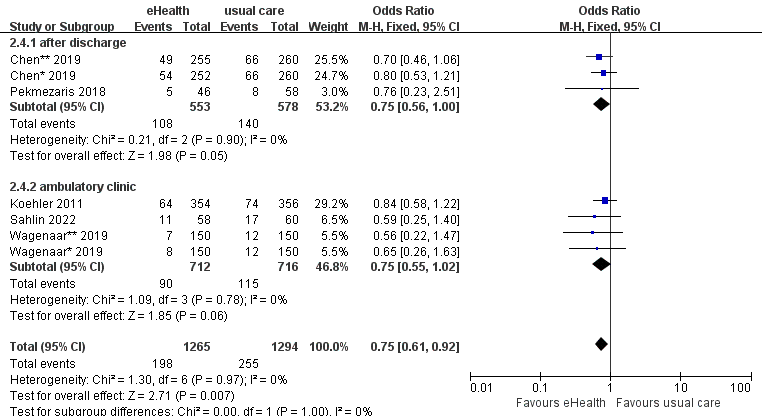

Supplement: Multimedia Appendix 2 [file jmir_v24i9e38697_app2.docx]
