# Supplementary material for: Effectiveness of eHealth Self-management Interventions in Patients With Heart Failure: Systematic Review and Meta-analysis
Source: J Med Internet Res. 2022 Sep 26;24(9):e38697. doi: 10.2196/38697 (PMC9555330; doi:10.2196/38697)

**Multimedia Appendix 3**

Table S1. Sensitivity analysis of the influence of each study on the pooled estimates. The leave-one-out approach was used.

(A) all-cause mortality

| Removed study | estimate | se | zval | pval | ci.lb | ci.ub | Q | Qp | I2 | H2 |
| --- | --- | --- | --- | --- | --- | --- | --- | --- | --- | --- |
| Çavuşoğlu 2016 | -0.1966 | 0.0832 | -2.3616 | 0.0182 | -0.3597 | -0.0334 | 22.8048 | 0.0441 | 42.9944 | 1.7542 |
| Chen** 2019 | -0.1775 | 0.0847 | -2.097 | 0.036 | -0.3435 | -0.0116 | 23.4273 | 0.0368 | 44.5091 | 1.8021 |
| Chen* 2019 | -0.1943 | 0.0849 | -2.287 | 0.0222 | -0.3608 | -0.0278 | 23.2493 | 0.0388 | 44.0842 | 1.7884 |
| Dendale 2011 | -0.1536 | 0.0825 | -1.8613 | 0.0627 | -0.3153 | 0.0081 | 19.1804 | 0.1176 | 32.2226 | 1.4754 |
| Galinier 2020 | -0.2266 | 0.0936 | -2.42 | 0.0155 | -0.4101 | -0.0431 | 22.5802 | 0.047 | 42.4275 | 1.7369 |
| Hindricks 2014 | -0.1337 | 0.0838 | -1.5946 | 0.1108 | -0.2979 | 0.0306 | 17.8847 | 0.1619 | 27.3121 | 1.3757 |
| Koehler 2011 | -0.2128 | 0.0886 | -2.4027 | 0.0163 | -0.3864 | -0.0392 | 22.7324 | 0.045 | 42.813 | 1.7486 |
| Koehler 2018 | -0.1191 | 0.0923 | -1.2906 | 0.1968 | -0.3 | 0.0618 | 21.3068 | 0.0671 | 38.9865 | 1.639 |
| Melin 2018 | -0.181 | 0.0819 | -2.2088 | 0.0272 | -0.3415 | -0.0204 | 23.4442 | 0.0366 | 44.5493 | 1.8034 |
| Pedone 2015 | -0.1704 | 0.0821 | -2.0767 | 0.0378 | -0.3313 | -0.0096 | 22.0289 | 0.0549 | 40.9865 | 1.6945 |
| Ritchie 2016 | -0.1828 | 0.0821 | -2.2272 | 0.0259 | -0.3436 | -0.0219 | 23.4786 | 0.0363 | 44.6304 | 1.806 |
| Sahlin 2022 | -0.186 | 0.0821 | -2.2651 | 0.0235 | -0.3469 | -0.025 | 23.3693 | 0.0375 | 44.3714 | 1.7976 |
| Seto 2012 | -0.1935 | 0.0817 | -2.3684 | 0.0179 | -0.3537 | -0.0334 | 21.423 | 0.065 | 39.3175 | 1.6479 |
| Wagenaar** 2019 | -0.1997 | 0.0823 | -2.4262 | 0.0153 | -0.361 | -0.0384 | 21.3551 | 0.0662 | 39.1247 | 1.6427 |
| Wagenaar* 2019 | -0.2109 | 0.0825 | -2.555 | 0.0106 | -0.3727 | -0.0491 | 19.0614 | 0.1212 | 31.7992 | 1.4663 |

(B) cardiovascular mortality

| Removed study | estimate | se | zval | pval | ci.lb | ci.ub | Q | Qp | I2 | H2 |
| --- | --- | --- | --- | --- | --- | --- | --- | --- | --- | --- |
| Çavuşoğlu 2016 | -0.2925 | 0.1184 | -2.4700 | 0.0135 | -0.5246 | -0.0604 | 6.9246 | 0.3279 | 13.3520 | 1.1541 |
| Chen** 2019 | -0.3056 | 0.1232 | -2.4798 | 0.0131 | -0.5471 | -0.0641 | 7.0976 | 0.3119 | 15.4648 | 1.1829 |
| Chen* 2019 | -0.3338 | 0.1240 | -2.6913 | 0.0071 | -0.5769 | -0.0907 | 6.6753 | 0.3519 | 10.1169 | 1.1126 |
| Hindricks 2014 | -0.2393 | 0.1204 | -1.9875 | 0.0469 | -0.4752 | -0.0033 | 4.0371 | 0.6717 | 0.0000 | 0.6728 |
| Koehler 2011 | -0.3539 | 0.1332 | -2.6566 | 0.0079 | -0.6149 | -0.0928 | 6.5422 | 0.3653 | 8.2876 | 1.0904 |
| Koehler 2018 | -0.2506 | 0.1372 | -1.8272 | 0.0677 | -0.5194 | 0.0182 | 6.5694 | 0.3625 | 8.6674 | 1.0949 |
| Wagenaar** 2019 | -0.3099 | 0.1164 | -2.6624 | 0.0078 | -0.5380 | -0.0818 | 6.9626 | 0.3243 | 13.8251 | 1.1604 |
| Wagenaar* 2019 | -0.3415 | 0.1174 | -2.9088 | 0.0036 | -0.5717 | -0.1114 | 4.1996 | 0.6497 | 0.0000 | 0.6999 |

(C) all-cause readmission rate

| Removed study | | estimate | se | | zval | | pval | | ci.lb | | ci.ub | | Q | | Qp | | I2 | | H2 | |
| --- | --- | --- | --- | --- | --- | --- | --- | --- | --- | --- | --- | --- | --- | --- | --- | --- | --- | --- | --- | --- |
| Chen** 2019 | | -0.1479 | 0.0677 | | -2.1858 | | 0.0288 | | -0.2805 | | -0.0153 | | 25.1746 | | 0.0329 | | 44.3883 | | 1.7982 | |
| Chen* 2019 | | -0.1669 | 0.0677 | | -2.4645 | | 0.0137 | | -0.2996 | | -0.0342 | | 27.705 | | 0.0156 | | 49.4676 | | 1.9789 | |
| Dorsch 2021 | | -0.2 | 0.0641 | | -3.1175 | | 0.0018 | | -0.3257 | | -0.0743 | | 28.4036 | | 0.0126 | | 50.7105 | | 2.0288 | |
| Galinier 2020 | | -0.218 | 0.0726 | | -3.0022 | | 0.0027 | | -0.3603 | | -0.0757 | | 28.5718 | | 0.0119 | | 51.0007 | | 2.0408 | |
| Hale 2016 | | -0.1836 | 0.0637 | | -2.885 | | 0.0039 | | -0.3084 | | -0.0589 | | 25.7498 | | 0.0278 | | 45.6307 | | 1.8393 | |
| Koehler 2011 | -0.2695 | | 0.0702 | -3.8401 | | 0.0001 | | -0.407 | | -0.1319 | | 22.6046 | | 0.067 | | 38.0658 | | 1.6146 | |  |
| Melin 2018 | -0.1989 | | 0.064 | -3.1052 | | 0.0019 | | -0.3244 | | -0.0733 | | 28.4935 | | 0.0122 | | 50.866 | | 2.0352 | |  |
| Negarandeh 2019 | -0.1801 | | 0.064 | -2.8162 | | 0.0049 | | -0.3054 | | -0.0548 | | 26.3149 | | 0.0236 | | 46.7982 | | 1.8796 | |  |
| Pedone 2015 | -0.1696 | | 0.0641 | -2.6446 | | 0.0082 | | -0.2953 | | -0.0439 | | 22.3943 | | 0.0709 | | 37.4841 | | 1.5996 | |  |
| Pekmezaris 2018 | -0.2012 | | 0.0643 | -3.1296 | | 0.0018 | | -0.3272 | | -0.0752 | | 28.3299 | | 0.0129 | | 50.5823 | | 2.0236 | |  |
| Ritchie 2016 | -0.2072 | | 0.065 | -3.1888 | | 0.0014 | | -0.3346 | | -0.0798 | | 27.9551 | | 0.0144 | | 49.9196 | | 1.9968 | |  |
| Sahlin 2022 | -0.1836 | | 0.0644 | -2.8512 | | 0.0044 | | -0.3099 | | -0.0574 | | 28.2835 | | 0.013 | | 50.5011 | | 2.0202 | |  |
| Seto 2012 | -0.1834 | | 0.0639 | -2.8676 | | 0.0041 | | -0.3087 | | -0.058 | | 27.5495 | | 0.0163 | | 49.1824 | | 1.9678 | |  |
| Wagenaar** 2019 | -0.1883 | | 0.0659 | -2.8569 | | 0.0043 | | -0.3175 | | -0.0591 | | 28.9259 | | 0.0107 | | 51.6004 | | 2.0661 | |  |
| Wagenaar* 2019 | -0.2082 | | 0.066 | -3.155 | | 0.0016 | | -0.3376 | | -0.0789 | | 28.3137 | | 0.0129 | | 50.5539 | | 2.0224 | |  |
| Yanicelli 2021 | -0.189 | | 0.0635 | -2.9755 | | 0.0029 | | -0.3136 | | -0.0645 | | 28.0353 | | 0.0141 | | 50.0629 | | 2.0025 | |  |

(D)HF-related readmission rate

| Removed study | estimate | se | zval | pval | ci.lb | ci.ub | Q | Qp | I2 | H2 |
| --- | --- | --- | --- | --- | --- | --- | --- | --- | --- | --- |
| Chen** 2019 | -0.2452 | 0.0837 | -2.9305 | 0.0034 | -0.4092 | -0.0812 | 2.6519 | 0.9765 | 0.0000 | 0.2947 |
| Chen* 2019 | -0.2665 | 0.0839 | -3.1765 | 0.0015 | -0.431 | -0.1021 | 2.8635 | 0.9695 | 0.0000 | 0.3182 |
| Dorsch 2021 | -0.263 | 0.0789 | -3.3336 | 0.0009 | -0.4176 | -0.1084 | 2.854 | 0.9698 | 0.0000 | 0.3171 |
| Galinier 2020 | -0.2553 | 0.0937 | -2.726 | 0.0064 | -0.4389 | -0.0718 | 2.8894 | 0.9685 | 0.0000 | 0.321 |
| Hale 2016 | -0.2545 | 0.0781 | -3.2582 | 0.0011 | -0.4076 | -0.1014 | 2.02 | 0.9911 | 0.0000 | 0.2244 |
| Hindricks 2014 | -0.2841 | 0.083 | -3.4224 | 0.0006 | -0.4468 | -0.1214 | 2.2165 | 0.9876 | 0.0000 | 0.2463 |
| Koehler 2011 | -0.2778 | 0.0854 | -3.253 | 0.0011 | -0.4452 | -0.1104 | 2.6574 | 0.9763 | 0.0000 | 0.2953 |
| Pekmezaris 2018 | -0.2601 | 0.0785 | -3.3112 | 0.0009 | -0.414 | -0.1061 | 2.9005 | 0.9681 | 0.0000 | 0.3223 |
| Sahlin 2022 | -0.2516 | 0.0792 | -3.1791 | 0.0015 | -0.4068 | -0.0965 | 2.5261 | 0.9802 | 0.0000 | 0.2807 |
| Wagenaar** 2019 | -0.2518 | 0.0789 | -3.191 | 0.0014 | -0.4065 | -0.0972 | 2.475 | 0.9815 | 0.0000 | 0.275 |
| Wagenaar* 2019 | -0.2553 | 0.079 | -3.2323 | 0.0012 | -0.4101 | -0.1005 | 2.7589 | 0.9731 | 0.0000 | 0.3065 |

**estimate:** estimated (average) outcomes.

**se:** corresponding standard errors.

**zval:** corresponding test statistics.

**pval:** corresponding p-values.

**ci.lb:** lower bounds of the confidence intervals.

**ci.ub:** upper bounds of the confidence intervals.

**Q:** test statistics for the test of heterogeneity.

**Qp:** corresponding p-values.

**tau2:** estimated amount of heterogeneity (only for random-effects models).

**I^2^:** values of I^2^.

**H^2^:** values of H^2^.

Figure S5. Sensitivity analysis of the influence of each study on the pooled estimate. The leave-one-out approach was used, which calculates the following parameters for each study: externally standardized residual, DFFITS value, Cook's distance, covariance ratio, the leave-one-out amount of (residual) heterogeneity, the leave-one-out test statistic of the test for (residual) heterogeneity, DFBETAS value(s).

(A) all-cause mortality


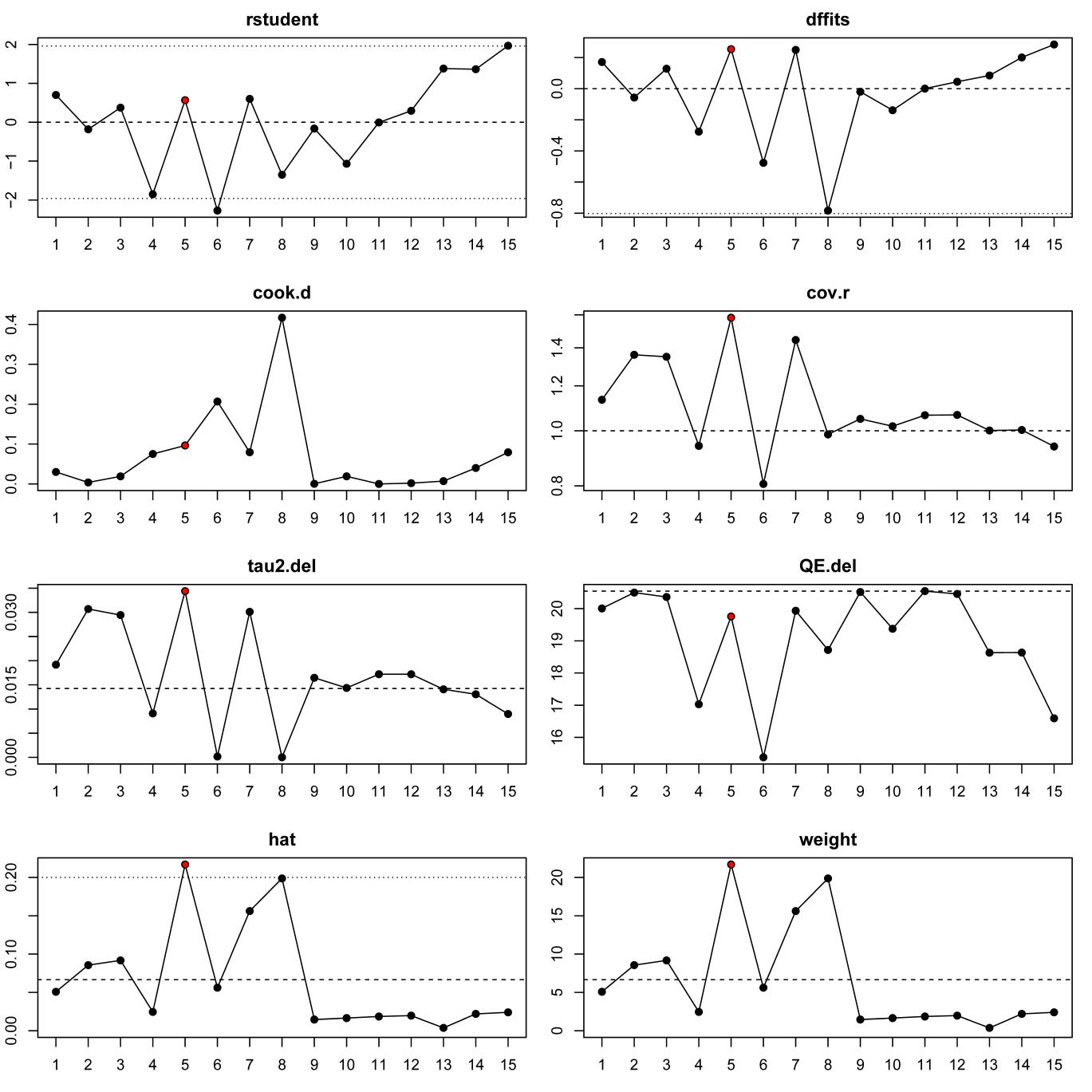


(B) cardiovascular mortality


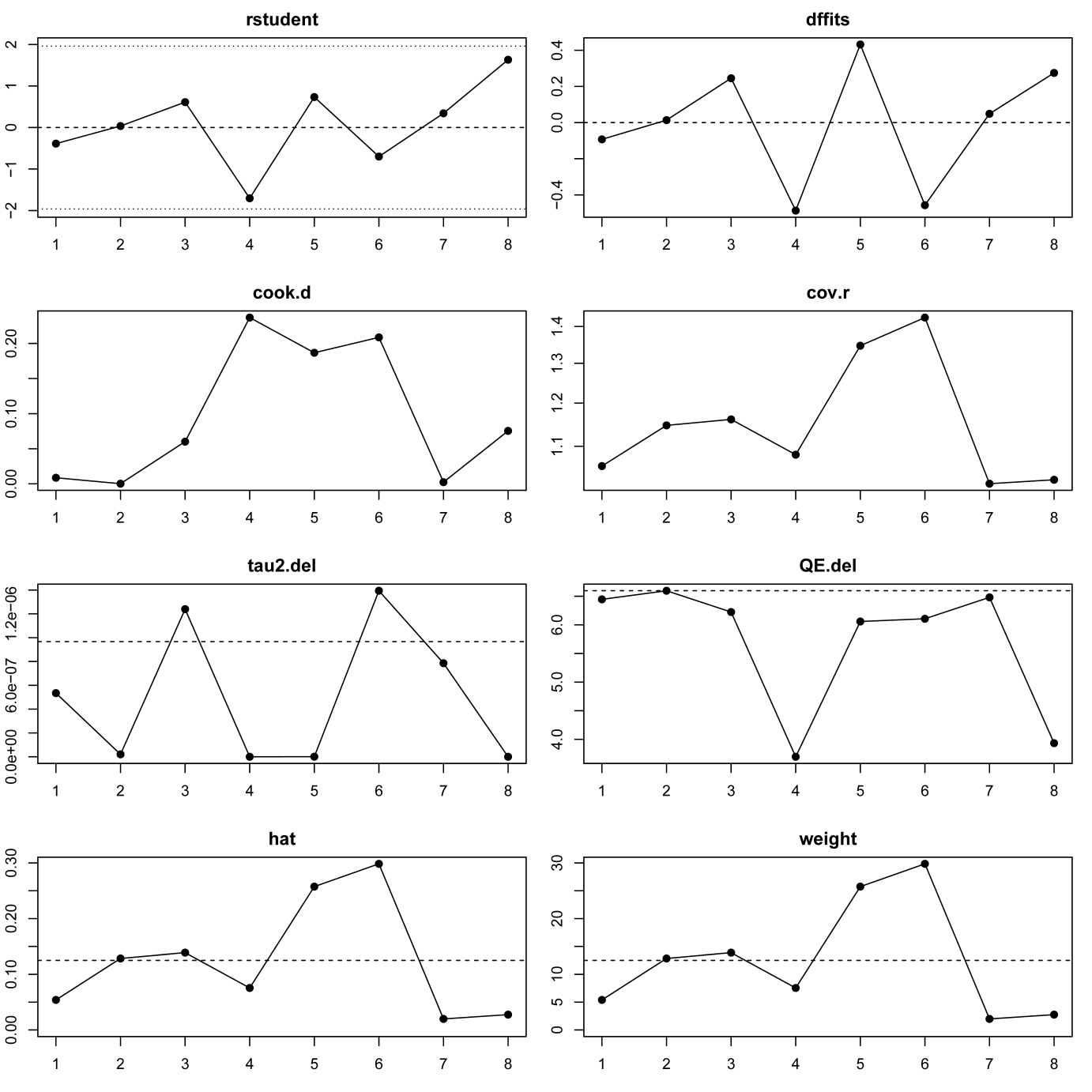


(C) all-cause readmission rate





(D)HF-related readmission rate


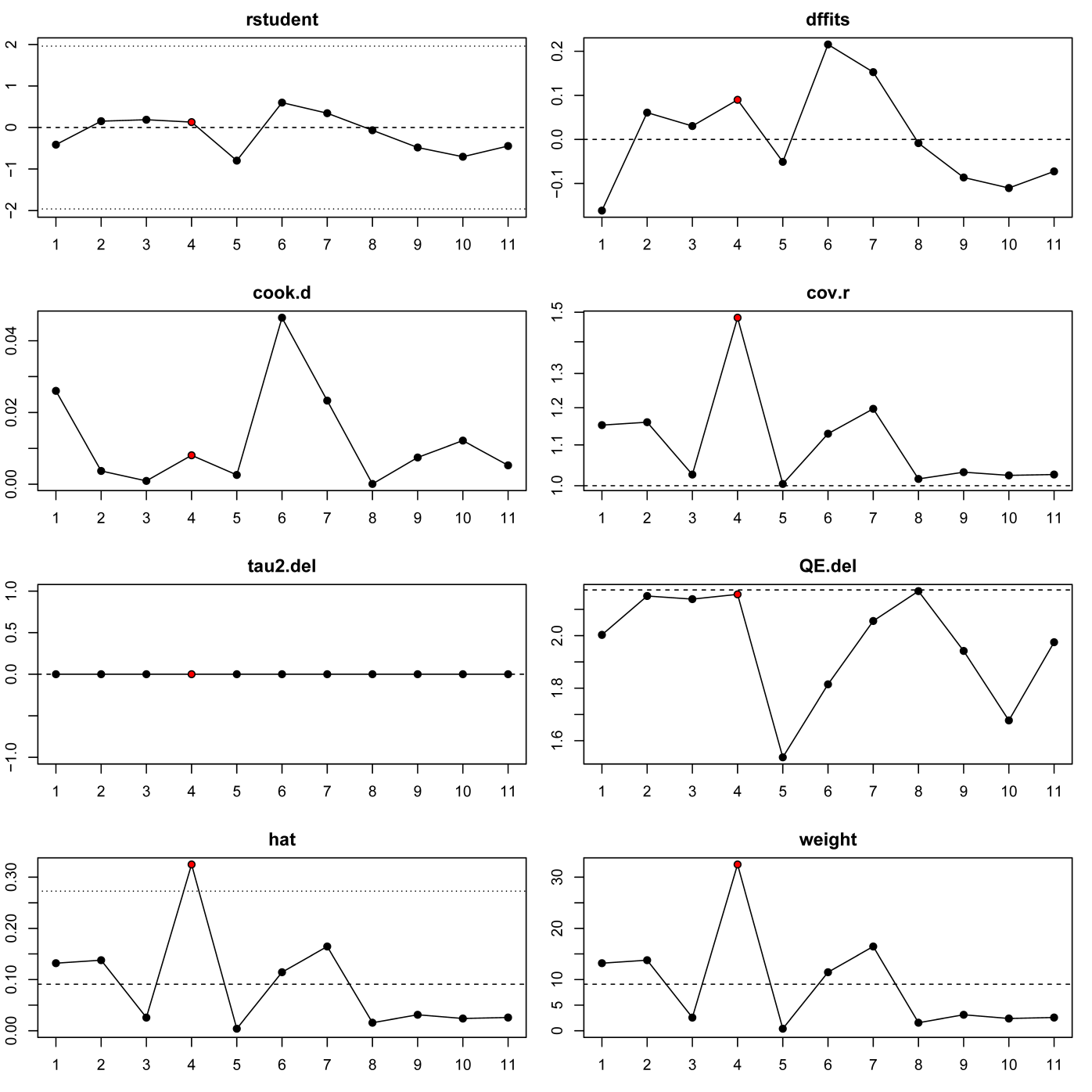

Supplement: Multimedia Appendix 3 [file jmir_v24i9e38697_app3.docx]
