# Supplementary material for: Effectiveness of eHealth Self-management Interventions in Patients With Heart Failure: Systematic Review and Meta-analysis
Source: J Med Internet Res. 2022 Sep 26;24(9):e38697. doi: 10.2196/38697 (PMC9555330; doi:10.2196/38697)
Supplement: Multimedia Appendix 4 [file jmir_v24i9e38697_app4.docx]

**Multimedia Appendix 4**

Figure S6. Funnel plots of all-cause mortality.


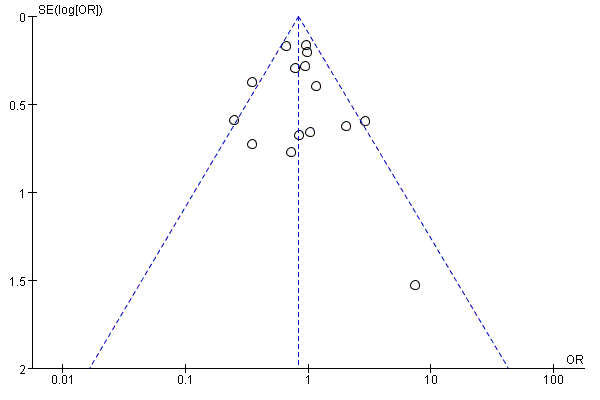


Linear regression test of funnel plot asymmetry

Test result: t = 0.34, df = 13, p-value = 0.7395

Figure S7. Funnel plots of cardiovascular mortality.


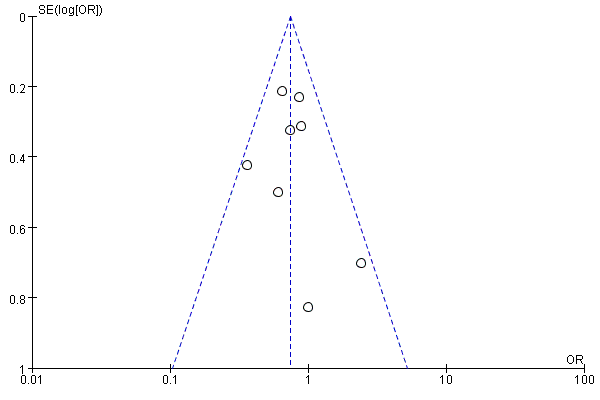


Linear regression test of funnel plot asymmetry

Test result: t = 0.52, df = 6, p-value = 0.6185

Figure S8. Funnel plots of all-cause readmission.


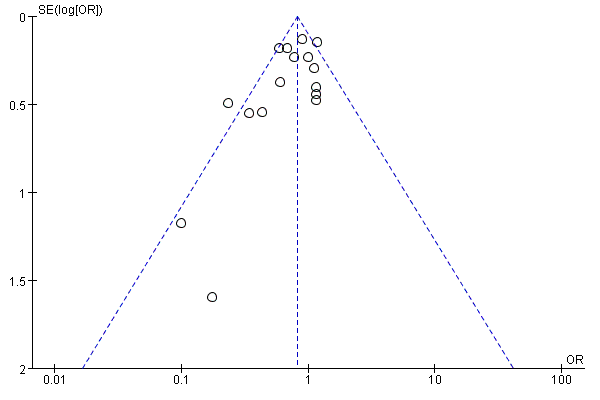


Linear regression test of funnel plot asymmetry

Test result: t = -2.01, df = 14, p-value = 0.0639

Figure S9. Funnel plots of HF-related readmission rate.


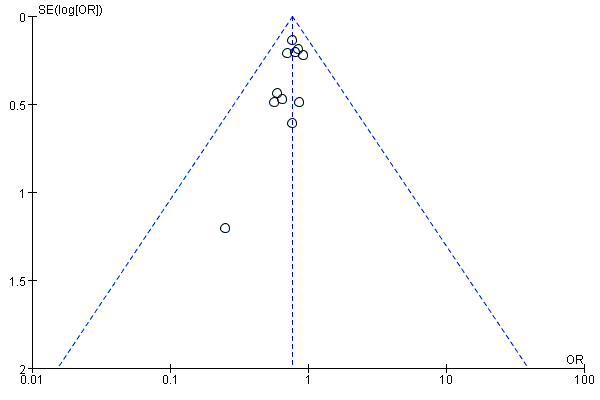


Linear regression test of funnel plot asymmetry

Test result: t = -2.00, df = 9, p-value = 0.0771
